# Epigenetics as a versatile regulator of fibrosis

**DOI:** 10.1186/s12967-023-04018-5

**Published:** 2023-03-02

**Authors:** Yangdan Liu, Dongsheng Wen, Chiakang Ho, Li Yu, Danning Zheng, Steven O’Reilly, Ya Gao, Qingfeng Li, Yifan Zhang

**Affiliations:** 1 Department of Plastic & Reconstructive Surgery, School of Medicine, Shanghai Ninth People’s Hospital, Shanghai Jiao Tong University, 639 Zhizaoju Road, Shanghai, 200011 China; 2 STipe Therapeutics, Aarhus, Denmark

**Keywords:** Fibrosis, Epigenetics, Chromatin remodeling, Histone modification, DNA methylation, Noncoding RNA

## Abstract

Fibrosis, a process caused by excessive deposition of extracellular matrix (ECM), is a common cause and outcome of organ failure and even death. Researchers have made many efforts to understand the mechanism of fibrogenesis and to develop therapeutic strategies; yet, the outcome remains unsatisfactory. In recent years, advances in epigenetics, including chromatin remodeling, histone modification, DNA methylation, and noncoding RNA (ncRNA), have provided more insights into the fibrotic process and have suggested the possibility of novel therapy for organ fibrosis. In this review, we summarize the current research on the epigenetic mechanisms involved in organ fibrosis and their possible clinical applications.

**Graphical Abstract:**

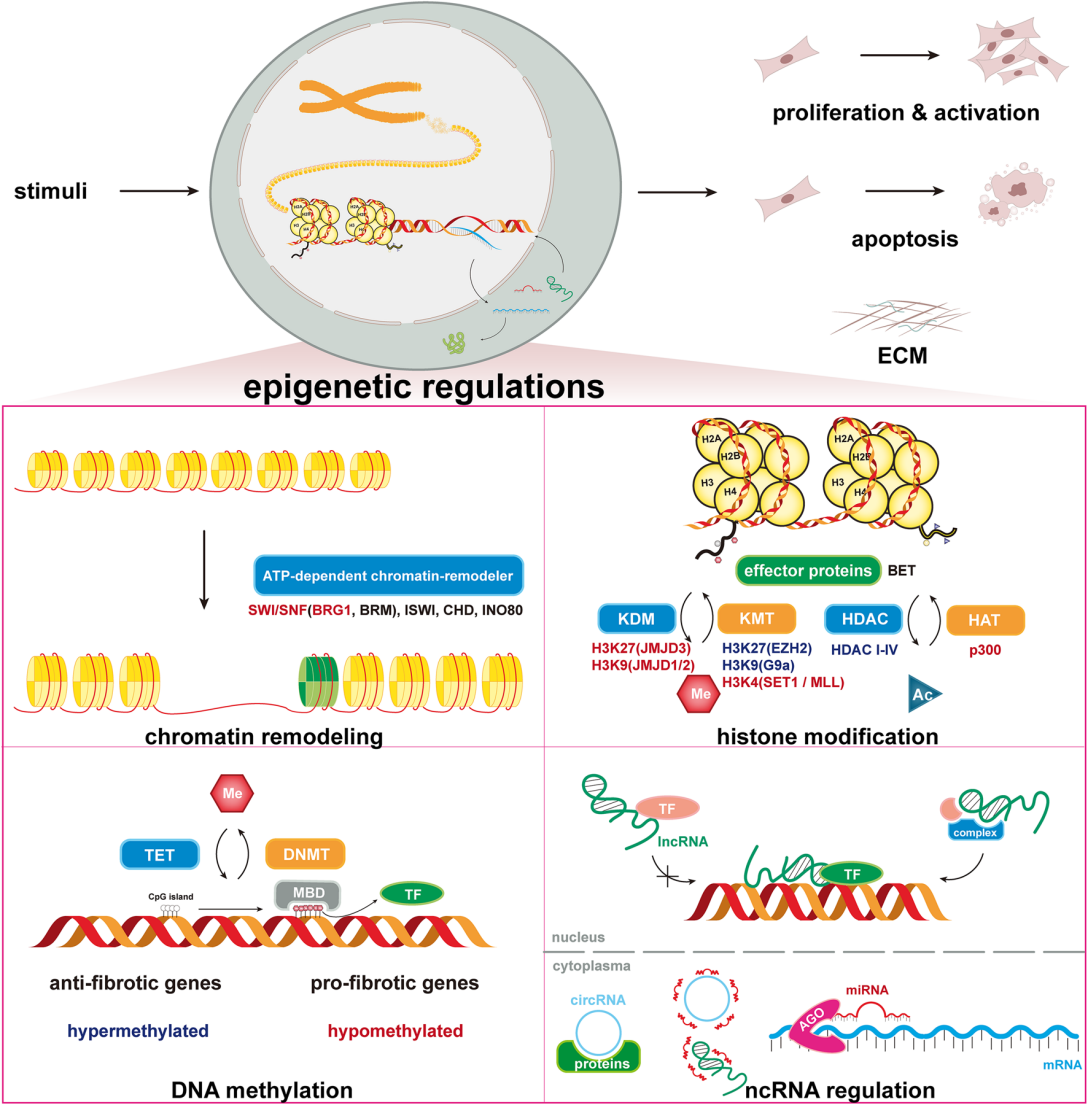

**Supplementary Information:**

The online version contains supplementary material available at 10.1186/s12967-023-04018-5.

## Background

Fibrosis is a reparative or reactive process characterized by the formation and deposition of excess fibrous connective tissue that results in progressive architectural remodeling in nearly all tissues and organs, including the liver, kidney, lung, heart, and skin [1]. In the different organs, specific clinical disease models present the initiation and progression of fibrosis (Table 1). Although fibrosis in different organs does have some organ-independent mechanisms, it mainly shares core process. The significant phases of fibrogenesis include (1) parenchymal cells destruction and associated inflammatory response (2) activation of fibrogenic effector cells, and (3) elaboration and dynamic deposition of ECM proteins [2]. In the process of inflammatory response, the local and invading immune cells produce a large variety of cytokines and chemokines that lead the transit of mesenchymal cells and other cells to myofibroblasts, which have the capacity to produce ECM and to further increase production of pro-inflammatory cytokines, chemokines, and angiogenic factors [3]. The active soluble mediators mentioned above are key effectors activating the downstream signaling pathways, including transforming growth factor-β (TGF-β) [4], platelet-derived growth factor (PDGF) [5], monocyte chemoattractant protein-1(MCP-1), Interleukins (IL-1β, IL-6, IL-13, IL-31 and IL-33).

**Table 1:** Common clinical disease models and animal models in organ fibrosis

Organ	Clinical disease models	Animal models
Lung	Idiopathic pulmonary fibrosis (IPF), radiation induced pulmonary fibrosis	Bleomycin induced pulmonary fibrosis
Heart	Myocardial infarction (MI) cardiomyopathy, chronic heart failure	MI induced cardiac fibrosis, transverse aortic constriction (TAC) induced cardiac fibrosis, Ang-II induced cardiac fibrosis
Liver	Non-alcoholic fatty liver disease (NAFLD), alcoholic liver disease (ALD) viral hepatitis	CCl4 induced liver fibrosis, thioacetamide (TAA) induced liver fibrosis, bile duct ligation (BDL) induced liver fibrosis
Kidney	Chronic kidney disease (CKD) easpecially diabetic and hypertrophic nephropathy	Ischemia–reperfusion injury (IRI) induced renal fibrosis, unilateral ureteral obstruction (UUO) induced renal fibrosis
Skin	Systemic Sclerosis (SSc), hypertrophic scar and keloid	Bleomycin induced skin fibrosis

In the past, fibrosis was once considered unidirectional, but a growing amount of evidence now suggests that fibrosis can be reversible under specific circumstances [6]. Until now, myofibroblast elimination and ECM degradation have been the two primary processes of fibrosis resolution [6]. Much effort has been made to study the regulation of fibrosis and to find a cure for this disease; however, the mechanisms behind the fibrotic process has not been thoroughly revealed, and no affirmative therapies have been approved. In recent decades, a number of studies focusing on epigenetic modifications have emerged, providing mechanistic insight into the occurrence and treatment of various diseases, such as cancer, neurological disease, and autoimmune disease [7–12], epigenetics seems to explain the reversible process and the environment impact on the pathologic process of diseases by being rapid and reversible.

The gene expression process, a highly conserved process in which a genotype gives rise to a phenotype, is well established and consists of unwinding and exposure of the DNA helix, transcription, RNA splicing, translation, and posttranslational modification. Each step of this process is under the precise control of epigenetic factors. First, chromatin remodeling can move, remove or alter nucleosomes by the action of chromatin remodeling complexes, a group of adenosine triphosphate (ATP)-powered protein complexes. Then, histone modifications open DNA-histone interactions as covalent posttranslational modifications of amino acids near the N-terminal ends of histone proteins [13]. After that, DNA methylation on the 5^th^ carbon of cytosine blocks the binding of transcription factors (TFs) by occupying the major groove of DNA. These two epigenetic regulations change the accessibility of DNA to TFs, thus influencing the subsequent transcription process [14]. Noncoding RNAs (ncRNAs), including both short and long ncRNAs are, to some extent, associated with epigenetic regulation. Their epigenetic functions are completed by regulating the expression or recruitment of proteins in the above epigenetic modification process [15, 16]. Each step of gene expression can be modified by epigenetics, thus leading to changes in downstream protein expression, function, and phenotype.

In this review, we provide a detailed and updated review of epigenetics in fibrosis, from mechanism to clinical practice, with the hope of offering a comprehensive understanding of fibrogenesis and its treatments with regard to epigenetics. We mainly focus on epigenetic modifications in fibrotic diseases to clarify the fundamental mechanism, classify the downstream pathways involved and develop potential therapies of fibrosis.

## From genetics to epigenetics in organ fibrosis

Previous research has significantly increased our understanding of genetic susceptibility to fibrotic diseases. Sequence variants in genes for surfactant proteins (SFTPC, SFTPA1, SFTPA2, ABCA3), polymorphisms in MUC5B or TOLLIP, and mutations in telomere genes (TERT, TERC, PARN, and RTEL1) are associated with an increased risk of idiopathic pulmonary fibrosis (IPF) [17, 18]. Polymorphisms in HLA genes (HLA-DQA1, HLA-DQB1, HLA-DPB1, HLA-DRB1) have been linked to SSc susceptibility, while immune-related genes (e.g., IRF genes) are also SSc drivers [19, 20]. Susceptibility loci in NAFLD, on the other hand, regulate lipid metabolism and promote hepatic lipid accumulation and toxicity [21].

However, the concordance rate for some fibrotic diseases in monozygotic twins is low and that common genetic variants are not always observed in patients, indicating that genetic predisposition is insufficient to explain disease development and suggesting a potential role of epigenetics as the missing link that connects the environmental exposure to disease development.

Interestingly, epigenetics and genetics are inextricably linked. On the one hand, sequence polymorphisms can influence epigenomic landscapes, and epigenetic factors are frequently mutated in diseases. Epigenetic mechanisms, on the other hand, regulate genome stability and mutability. In IPF, rs35705950, a MUC5B promoter variant, was discovered to disrupt a CpG motif, resulting in a significant increase in MUC5B expression by inhibiting DNA methylation [22]. With ATAC-seq analysis, a recent study confirmed that the rs35705950 resides within an enhancer that is subject to epigenetic remodeling [23]. It has been suggested that the I148M mutation in NAFLD may regulate PNPLA3 gene expression through methylation at specific loci [24]. Furthermore, telomere shortening is thought to be triggered in part by epigenetic mechanisms [25].

## Chromatin remodeling

Chromatin remodeling is a process using the energy of ATP hydrolysis to destabilize, move, or restructure nucleosomes. Chromatin remodeling complexes contain four different families, SWI/SNF, ISWI, CHD, and INO80, but share a relatively conserved ATPase domain. The result of their actions on nucleosome arrays can be classified into two categories: site exposure, where the site for DNA-binding protein (DBP) becomes accessible, and altered composition, where the nucleosome content is modified by histone variant replacement or eviction [26].

**Fig. 1:**
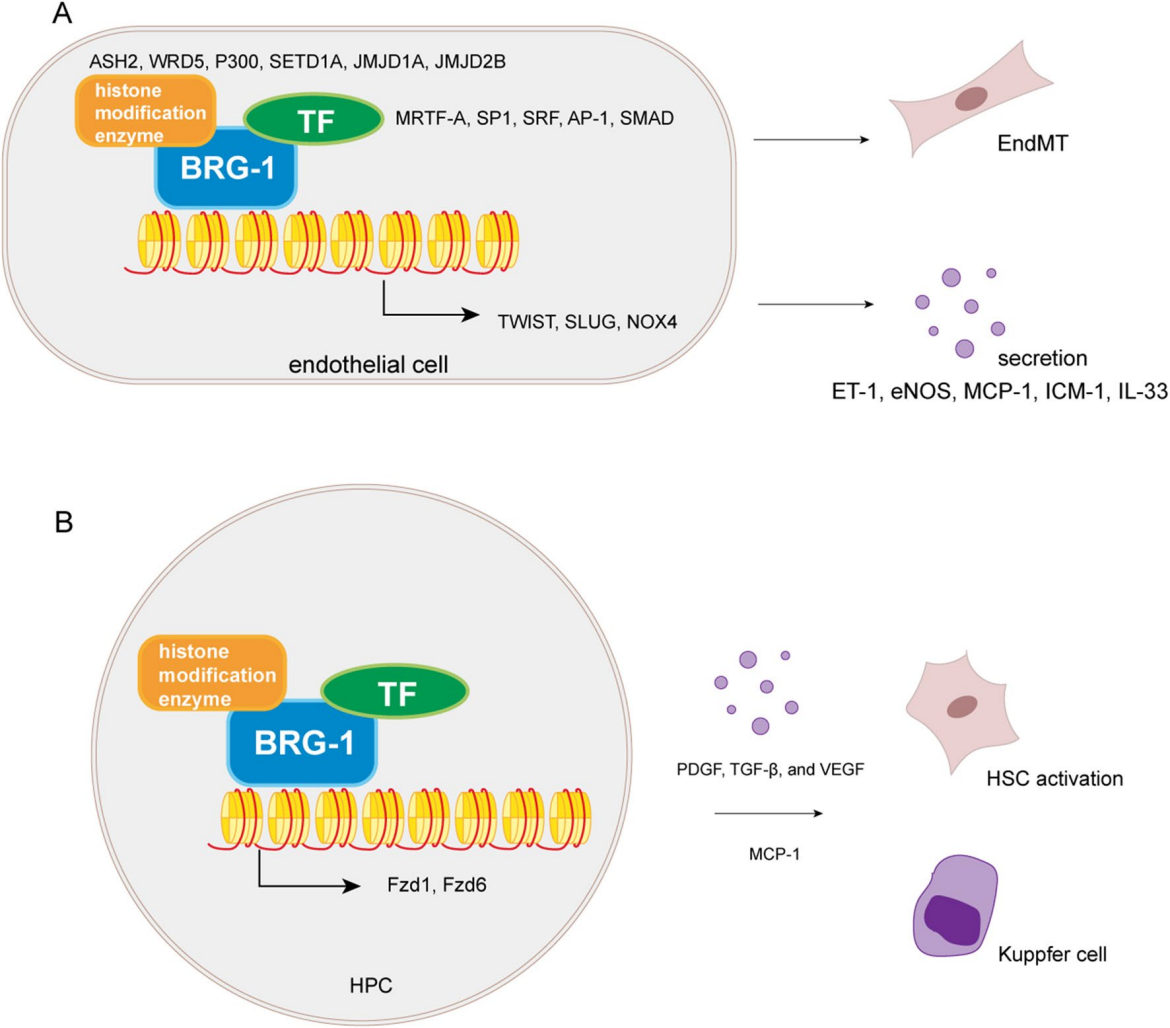
Roles of Chromatin remodeling complexes in fibrosis. **A** BRG1 acts with TFs and histone modification enzymes to regulate EndMT in fibrosis. **B** BRG1 promotes HPC activation in liver fibrosis by activating HSC and Kupffer cells

Chromatin remodelers are poorly studied in fibrosis since the mechanism of Chromatin remodeling is not clear. The SWI/SNF family, the most studied group of chromatin remodelers, contains two catalytic ATPase subunits, BRM (Brahma/SMARCA2) and BRG1 (Brahma-related gene 1/SMARCA4)[27]. A series of studies have already revealed the role of BRG1 in endothelial cells (Fig. 1A, Table 2). By interacting with TFs (eg. MRTF-A, SP-1, SRF, AP-1, SMAD3) or histone modification enzymes(eg. ASH2, p300, KDM3A), together they bind to the promoters of varies genes and regulates their expression [28–30]. However, the exact order of these factors binding to the promoter has not been extensively studied. These epigenetic regulators mainly play a role in endothelial-to-mesenchymal transition (EndMT) through the decreased expression of endothelial markers and increased expression of mesenchymal markers by regulating key transcription factors (eg. TWIST, SLUG) [28, 31], or through the regulation of Reactive Oxygen Species (ROS) pathway by NADPH oxidase 4 (NOX4) indirectly [32]. In addition to the EndMT process, BRG1 has also been found to act in the regulation of mediators secreted by endothelial cells, such as Endothelin 1(ET-1), endothelial nitric oxide synthase (eNOS), MCP-1 or cell adhesion molecule intercellular cell adhesion molecule-1 (ICAM-1), which are key factors regulating the chemotaxis and adhesion of macrophages or neutrophils in the inflammatory response [30, 33]. Other mediators, such as IL-33 has also been found to be regulated by BRG1 and promote fibrosis through the TGF-β pathway [34].

**Table 2:** Mechanisms of chromatin remodeler BRG1 in organ fibrosis

Organ	Cell	Co-factors	Target genes	Transcriptional regulation	Effect on fibrosis	Refs.
Heart	Endothelial cell	Sp1, SRF	SNAI2, COL1A2	Activation	Pro	[28]
Heart	Endothelial cell	Ash2, MRTF-A	ET-1	Repression	Pro	[29]
Kidney	Endothelial cell	Smad3	IL-33	Activation	Pro	[34]
Liver	Endothelial cell	Sp1	CAV-1	Activation	Pro	[30]
Liver	Endothelial cell	HIF-1α	TWIST	Repression	Pro	[31]
Liver	Endothelial cell	Smad3, AP-1	NOX4	Repression	Pro	[32]
Liver	Hepatic stellate cell	HIF-1α, p300, ASH2, KDM3A	α-SMA, COL1A1	Activation	Pro	[36]

Other studies have also found that BRG1 in fibroblasts, hepatic stellate cells, renal epithelial cells, etc. regulate fibrosis through the TGF-β pathway or the Wnt pathway [35, 36]. A recent clinical study has also shown that hepatic progenitor cells (HPCs) activation is highly associated with liver fibrogenesis, which are always highly correlated with BRG1 expression (Fig. 1B, Table 2). HPCs are activated possibly through the Wnt pathway, and directly activate hepatic stellate cells (HSCs) by producing PDGF, TGF-β, and vascular endothelial growth factor A (VEGF) or they can also recruit Kupffer cells by MCP-1 [37].

## Histone modification

Histones are proteins that provide structural support to form nucleosomes. Each nucleosome consists of two identical subunits, and each subunit contains four histones: H2A, H2B, H3, and H4, which are also recognized as core histones. H1 is located at the gate of core histones and functions to link and stabilize two nucleosomes. Histones pack DNA into chromatin and are thus crucial in the transcription of DNA by deciding which segment is exposed and can be accessed [38, 39].

Four types of histone regulation that have been identified in recent decades, acetylation [40], methylation [41], phosphorylation [42], and ubiquitination [43], are universally established, while N-acetyl glucosamine glycosylation, citrullination, crotonylation, and isomerization have only been recently reported [13, 44, 45].

### Histone acetylation and deacetylation

The acetylation of histones is one of the earliest identified histone modifications. Acetylation negatively charges the lysine residues of the N-terminal histone tail to repel the negatively charged DNA and causes chromatin structure relaxation. The opened chromatin conformation allows transcription factors to bind and gene expression to increase [46, 47]. Acetyl can be added to the lysine residues of histones H3 and H4 by histone acetylases (HATs) and can be removed by deacetylases (HDACs) [48, 49].

#### Histone acetylase

HAT consists of three major families: general control nonderepressible 5 (Gcn5)-related N-acetyltransferases (GNATs), p300/CBP, and MYST proteins [50–52], among which p300/CBP is most important in fibrosis; its mechanism has been elaborated, and corresponding inhibitors have been discovered.

**Fig. 2:**
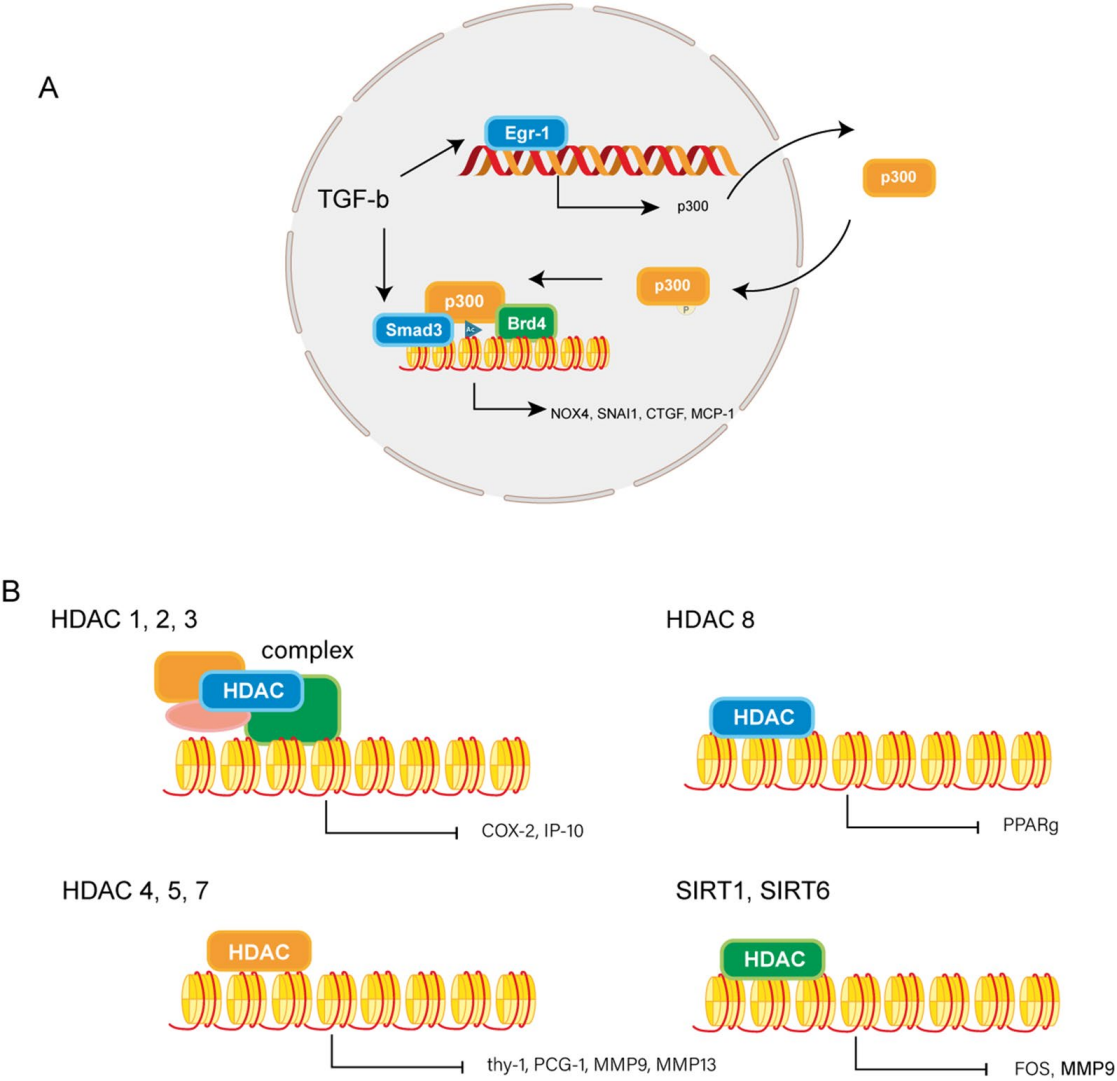
Roles of histone acetylation in fibrosis. **A** Interactions between Histone acetylase p300 and TGF-β signaling in fibrosis. **B** Roles of different classes of HDACs in fibrosis in different signaling pathways

The role of p300 in fibrosis has been verified in multiple studies (Fig. 2A, Table 3). P300, as a histone acetylase, can induce histone acetylation of MCP-1 [53], NOX4 [54], and other gene promoters to promote the fibrogenesis process in IPF. In the process of p300 regulation, in addition to the cis mechanism (charge effects), a type of protein plays a vital role as a “reader” in the trans mechanism [55]. Bromodomain-containing protein 4 (Brd4) is a member of the bromodomain and extraterminal (BET) family of proteins, which function as epigenetic “readers” of acetylated lysine groups on histones. The vital roles of Brd4 and its inhibitor JQ1 have been proven in the epigenetic regulation of p300 in various profibrotic genes, such as NOX4, snail family transcriptional repressor 1 (SNAI1), and CTGF in IPF and myocardial infarction (MI) induced cardiac fibrosis [54, 56–59]. P300 functions in the TGF-β signaling pathway as a coactivator with Smad3. First, TGF-β regulates p300 expression by Early Growth Response 1 (EGR1) [60, 61] and can regulate the translocation of p300 through posttranslational modification; for example, the phosphorylation of p300 by AKT signaling has been reported to induce its translocation to the nucleus in liver fibrosis [62]. Nucleic p300 then increases the synthesis of collagens by interacting with TGF-β-activated Smad3 on the collagen gene promoter.

**Table 3:** Epigenetic mechanisms of histone modifications in organ fibrosis

Histone modification	Function	Molecule	Target genes	Transcriptional regulation	Effect on fibrosis	Organ	Refs.
Acetylation	Acetylase	P300	CCL2	Activation	Pro	Lung	[53]
			NOX4	Activation	Pro	Lung	[54]
	Reader	BRD4	SNAI1, ZEB1, TWIST1	Activation	Pro	Lung	[57]
			NOX4	Activation	Pro	Lung	[54]
			SERTAD4	Activation	Pro	Heart	[59]
			NPPA, NPPB, CTGF	Activation	Pro	Heart	[58]
Deacetylation	Deacetylase (Class I HDACs)	HDAC1, HDAC2, HDAC3	COX-2	Repression	Pro	Lung	[78]
		HDAC1	COL1A1	Repression	Anti	Skin	[80]
		HDAC1	MAP2K3	Repression	Anti	Heart	[83]
		HDAC3	WIF-1	Repression	Pro	Skin	[81]
		HDAC8	PPARγ	Repression	Pro	Lung	[82]
	Deacetylase (Class II HDACs)	HDAC4, HDAC5, HDAC7	Thy-1	Repression	Pro	Lung	[84]
		HDAC4	SIRT1	Repression	Pro	Liver	[91]
		HDAC4	MMP9	Repression	Pro	Liver	[88]
		HDAC7	HGF	Repression	Pro	Liver	[89]
		HDAC7	PGC1α	Repression	Pro	Lung	[85]
	Deacetylase (Class III HDACs)	SIRT1	MMP9	Repression	Anti	Lung	[95]
		SIRT3	FOS	Repression	Anti	Heart	[93]
		SIRT6	IGR signaling related genes	Repression	Anti	Heart	[94]
Methylation	Methylase (H3K27)	EZH2	Smad7, PTEN	Repression	Pro	Kidney	[101]
		EZH2	CTGF, CCL2	Repression	Anti	Kidney	[105]
		EZH2	Dkk1	Repression	Pro	Liver	[102]
		EZH2	PPARγ	Repression	Pro	Liver	[103]
		/	FRA2	Repression	Anti	Skin	[104]
	Methylase (H3K9)	G9a	PGC1α	Repression	Pro	Lung	[112]
		G9a	IP-10	Repression	Pro	Lung	[79]
		/	Fas	Repression	Pro	Lung	[87]
		G9a	E-cadherin	Repression	Pro	Lung	[116]
		G9a	Klotho	Repression	Pro	Kidney	[114]
	Methylase (H3K27 & H3K9)	EZH2, G9a	CXCL10, COX-2	Repression	Pro	Lung	[109]
	Methylase (H3K4)	COMPASS	COL1A1, COL1A2, α-SMA	Activation	Pro	Liver	[127]
		COMPASS	COL1A1, COL1A2	Activation	Pro	Kidney	[126]
		SET7/9	TGF-β1	Activation	Pro	Liver	[128]
		SET7/9	COL1A1, CTGF, PAI-1	Activation	Pro	Kidney	[129]
		ASH1	COL1A1, COL1A2, α-SMA, TIMP1, TGF-β1	Activation	Pro	Kidney	[130]
	Demethylase (H3K27)	JMJD3(KDM6B)	CTGF, CCL2	Activation	Pro	Kidney	[105]
		JMJD3(KDM6B)	FRA2	Activation	Pro	Skin	[107]
		JMJD3(KDM6B)	XIAP, survivin	Activation	Anti	Lung	[106]
	Demethylase (H3K9)	JMJD1A(KDM3A)	YAP1, TGF-β2	Activation	Pro	Kidney	[118]
		JMJD1A(KDM3A)	CTGF	Activation	Pro	Kidney	[119]
		JMJD1A(KDM3A)	TIMP1	Activation	Pro	Heart	[120]
		JMJD1A(KDM3A)	PPARγ	Activation	Anti	Liver	[122]
		KDM4A/B/C	miR-29	Activation	Anti	Liver	[123]
		KDM4D	TLR4	Activation	Pro	Liver	[121]

Previous study has identified strategies that work against this profibrotic effect, a small molecule inhibitor, L002, has been found to mediate the suppression of the acetylase activity of p300 in fibroblasts, resulting in the repression of TGF-β-induced H3K9 acetylation, thus inhibiting myofibroblast differentiation and collagen synthesis in hypertrophic nephropathy [63]. p300 may interact with other epigenetic regulators. Sirtuins and other microRNAs, such as miR-200b, miR-132, and miR-133a, have been identified in fibrosis by regulating the expression of p300 [64–69].

#### Histone deacetylation

The acetyl of histone could be removed by deacetylation through a series of histone deacetylases, triggering a compact nucleosome structure and preventing active transcription. HDAC can be classified into four distinct groups based on its function, DNA sequence, and domain organization. Class I HDACs include HDAC1, HDAC2, HDAC3, and HDAC8, which are widely expressed and found mainly in the nucleus. Class IIa HDACs include HDAC4, 5, 7, and 9, while class IIb HDACs include HDAC6 and 10. These two classes are subdivided based on the number of catalytic domains the proteins possess. Class III HDACs include sirtuins (SIRTs) and nicotinamide adenosine-dependent (NAD) enzymes. Class IV HDACs contain only one member, HDAC11, which shares sequence domains with class I and class III HDACs. HDACs epigenetically alter the gene transcription process via the deacetylation of core histones. They increase the positive charges on histones and possibly strengthen histone-DNA interactions and repress transcription. However, whether HDACs can directly activate transcription and the exact detailed mechanisms by which they regulate transcription remain to be clarified [70].

HDACs can regulate fibrosis via fibroblast proliferation, senescence, and ECM production [71–74]. Several signaling pathways participate in fibrosis, including the TGF-β pathway, the Wnt pathway, and apoptosis signaling pathways [75]. In the following chapter, we will discuss the function of HDACs in regulating fibrosis in all these processes (Fig. 2B, Table 3).

HDAC1 and HDAC2 coexist to form Sin3, NuRD, and CoREST complexes [76]. HDAC3 can also interact with SMRT/NCoR to stimulate the enzymatic activity of HDAC3 [77]. These complexes have been found to be significant in fibrogenesis through the regulation of COX-2 and IP-10. The binding of the CoREST and mSin3a transcriptional corepressor complexes, as well as the NCoR complex with the COX-2 promoter, is markedly strengthened, resulting in the insufficient acetylation of histone H3 and H4 and weakening the binding of the transcription factors NF-κB, C/EBPβ, and CREB-1 to the COX-2 promoter, eventually leading to diminished COX-2 transcription in IPF [78]. The epigenetic regulation of IP-10 is almost the same [79]. COL1A1 and SMAD7 are also inhibited via the recruitment of repressor complexes comprising SP1, SIN3A, CoREST, LSD1, and HDAC1 to the promoter in systematic sclerosis (SSc) [80]. In addition, HDACs have been found to be recruited by transcription factors to the promoter. For example, HDAC3 is recruited by activating transcription factor 3 (AFT3) to the Wnt inhibitor factor 1 (WIF-1) promoter and inhibits WIF-1 expression in SSc, which induces COL1A1 expression [81]. In contrast to the findings above, some class I HDACs function independently in fibrotic pathways. HDAC8 inhibition at least partially represses TGF-β-induced fibrosis by increasing PPARγ gene transcription via restoration of H3K27 acetylation at the enhanced region and finally regulates CTGF, plasminogen activator inhibitor type 1 (PAI-1), and α-smooth muscle actin (α-SMA) expression in IPF [82]. However, some class I HDACs were also found to play an antifibrotic role in fibrosis. HDAC1 was recruited to the mitogen-activated protein kinase kinase 3 (MAP2K3) promoter by AFT3, resulting in MAP2K3 gene-associated histone deacetylation, thereby inhibiting MAP2K3 expression. MAPK signaling is then activated to inhibit profibrotic effects in SSc [83].

Recruitment of HDAC4, HDAC5, and HDAC7 promotes the deacetylation of H3 and H4 histones. They can enhance fibroblast proliferation by inhibiting thy-1 and promoting fibroblast activation through downregulation of peroxisome proliferator-activated receptorγ coactivator-1 (PGC-1) in IPF [84–86]. They can also inhibit fibroblast apoptosis by downregulating Fas signaling [87] and ECM degradation by inhibiting the expression of MMP9 in liver fibrosis [88]. Nucleocytoplasmic shuttling has been found in fibrosis. Nucleic HDAC7 can bind to the promoter of hepatocyte growth factor (HGF), which leads to the repression of HGF and induces liver fibrosis. Cylindromatosis (CYLD) was discovered to stimulate the export of HDAC7 to cytoplasm independently of the classic mechanism to ameliorate organ fibrosis [89]. HDAC7 has also been shown to regulate collagen and other ECM in systemic sclerosis fibroblasts and siRNA mediated depletion of HDAC7 reduced ECM in these cells [90]. Interestingly, HDAC4 was discovered to remove acetylated histones H3 and H4 from the SIRT1 promoter, and SIRT1 can thus deacetylate PPARγ to block fibroblast activation [91].

Class III HDAC and SIRT has been found to involve in nonhistone protein modifications in fibrosis and only a few studies have revealed its role in histone modifications [92]. SIRT6 can not only inhibit insulin-like growth factor (IGF) and resist fibroblast apoptosis through the Akt pathway but can also regulate the expression of the transcription factor FOS, regulating the transcription of fibrosis-related genes upstream [93, 94]. SIRT1 was found to induce ECM degradation by deacetylating histones on the MMP9 promoter, thereby suppressing its transcription in chronic obstructive pulmonary disease (COPD) [95].

The studies described above have illustrated the multifunctional and diverse mechanisms of HDACs by targeting various genes in the processes of fibroblast proliferation, apoptosis, ECM deposition, and degradation. Notably, HDACs act as fundamental regulators participating in epigenetic modifications, thus making them a generally recognized potential treatment target for fibrosis.

### Histone methylation

Compared to histone acetylation, methylation has been characterized as a more complex entity since distinct histone lysine residues may have various functions when methylated. Additionally, different densities of methylation on the same residue may vastly differ in their functions [96, 97]. Unlike acetylation, methylation does not change the histone charge, nor does it directly affect the histone-DNA interaction. Rather, methylation regulates gene expression through gene transcription, chromatin remodeling, and other epigenetic modifications [98, 99].

#### H3K27 methylation

Compared to histone acetylation, methylation has been characterized as a more complex entity since distinct histone lysine residues may have various functions when methylated. Additionally, different degrees of methylation on the same residue may vastly differ in their functions [96, 97]. Unlike acetylation, methylation does not change the histone charge, nor does it directly affect the histone-DNA interaction. Rather, methylation regulates gene expression through gene transcription, chromatin remodeling, and other epigenetic modifications [98, 99].

**Fig. 3:**
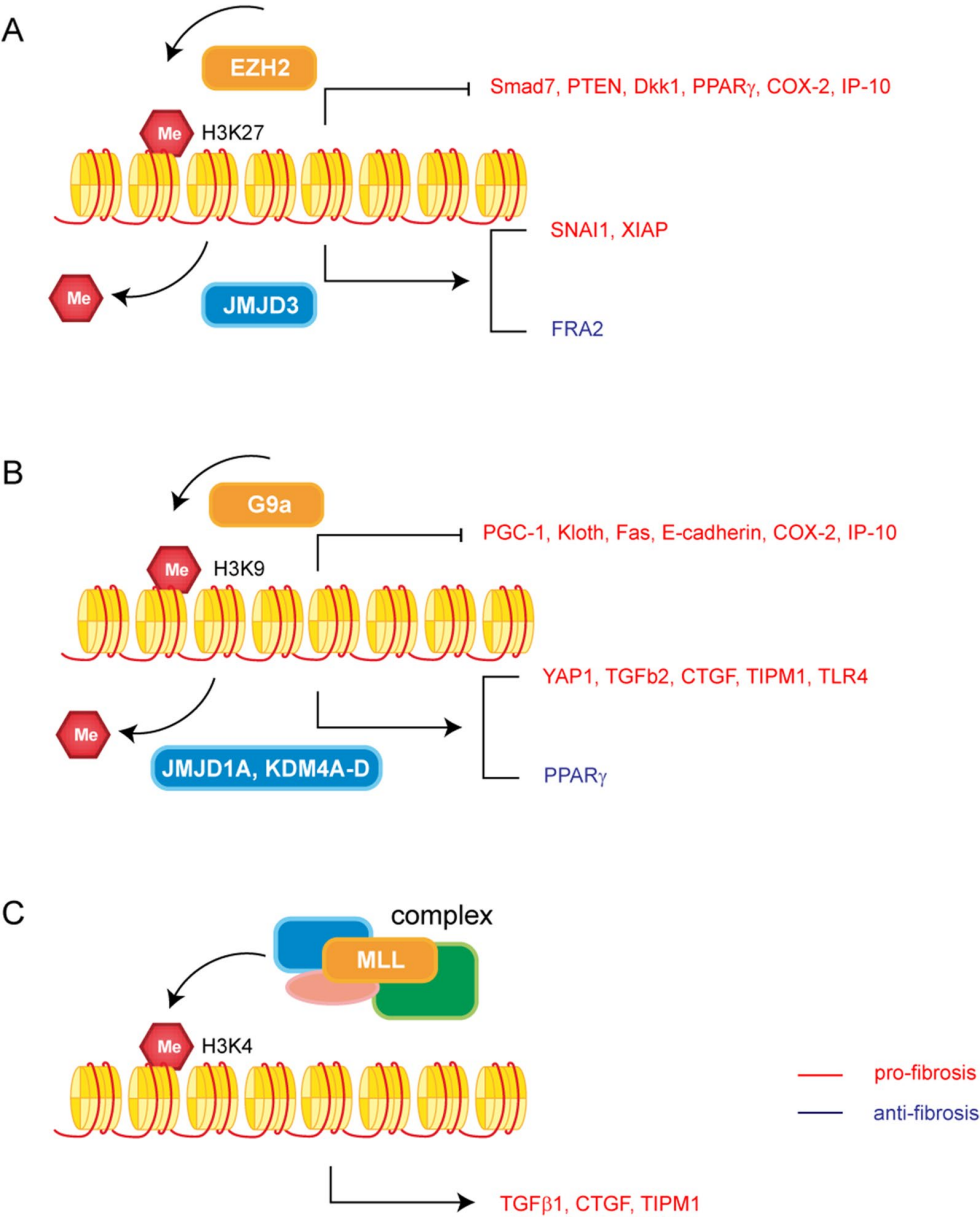
Roles of histone methylation in fibrosis. **A** Roles of H3K27 methylation in fibrosis. **B** Roles of H3K9 methylation in fibrosis. **C** Roles of H3K4 methylation in fibrosis

Enhancer of Zeste Homolog 2 (EZH2) is a histone methylase, together with embryonic ectoderm development (EED) and suppressor of zeste 12 (SUZ12), forming polycomb repressive complex 2 (PRC2) to mediate H3K27me3, which is essential for stable silencing (Fig. 3A, Table 3) [100]. EZH2 has been mostly found to regulate fibroblast proliferation, cell transdifferentiation, and ECM production and was found to be involved in various signaling pathways. Increased EZH2 caused downregulation of Smad7 and phosphatase and tensin homolog deleted on chromosome 10 (PTEN). As a result, the TGF-β/Smad3 signaling, EGFR, and PDGFR signaling pathways are activated, leading to the activation of fibroblasts and ECM deposition in diabetic nephropathy [101]. Epigenetic silencing of Dkk1 by EZH2 is a critical mechanism mediating HSC activation and fibrogenesis in liver fibrosis [102]. EZH2 has also been proven to mediate the transcriptional repression of the antifibrotic gene PPARγ [103]. Although in SSc. EZH2 inhibition actually enhanced fibrosis [104].

The demethylation of H3K27 was associated with Jumonji domain-containing protein D3 (JMJD3, encoded by KDM6B) and ubiquitously transcribed tetratricopeptide repeat on chromosome X (UTX, encoded by KDM6A). In recent studies, repression of EZH2 and activation of JMJD3 were demonstrated to work together in TGF-β-induced fibrosis, repressing H3K27me3 and thus increasing profibrotic gene expression [105]. The direct interaction of JMJD3 with promoter regions of X-linked inhibitor of apoptosis protein (XIAP) and survivin, which are members of the inhibitor of apoptosis protein (IAP) family, attends the resistance of fibroblasts to apoptosis [106]. Similar to EZH2, the paradoxical role of JMJD3 was discovered in skin fibrosis. Inhibition of JMJD3 ameliorated bleomycin-induced and topoI-induced fibrosis in well-tolerated doses, mechanically, inactivation of JMJD3 reduced the expression of fos-related antigen-2 (fra-2), a member of the AP1 family of transcription factors that has previously been shown to play a central role in the pathogenesis of SSc [107].

Significantly, different methylases can work together or interfere with other epigenetic factors, establishing a crosstalk network. A set of studies found that pulmonary fibrosis was regulated by G9a/EZH2-mediated H3K9me3/H3K27me3, interacting with DNA methylation in a bidirectional and mutually dependent manner to reinforce COX-2 and CXCL10 epigenetic silencing [108, 109]. DNA methylation has also been found to recruit histone methylase. It has been reported that DNA methylation plays a role in H3K9me since DNMT3a can recruit the histone methylase SUV39H1 using its PHD-like motif [110]. MeCP2 also recruits EZH2 to induce H3K27me and ultimately induce the transcriptional repression of PPARγ in organ fibrosis [103].

#### H3K9 methylation

In addition to EZH2, another methylase, G9a, catalyzes the methylation of H3K9, and this modification serves as a binding site for chromodomain protein heterochromatin protein 1 (HP1), thus generating local heterochromatin (Fig. 3B, Table 3) [111]. Either G9a or HP1 can inhibit the expression of gene PPARGC1A and promote fibrosis. PGC1a has been proved to inhibit fibrosis in multiple animal models, and is possibly related to mitochondrial metabolism [112]. A study found that G9a-induced H3K9me1 had a pivotal role in reducing Klotho expression, and Klotho appeared to be the primary mediator of antifibrotic effects through inhibition of the TGF-β1, Wnt, and other fibrosis-related signaling pathways [113, 114]. H3K9me3 is responsible for the decreased expression of the death receptor Fas and resistance to Fas-mediated apoptosis in fibroblasts [87]. Moreover, in radiation-induced pulmonary fibrosis, enrichment of H3K9me2/3 has been found in E-cadherin promoter in epithelial cells, and its positive regulation of EMT can be inhibited by the G9a inhibitor BIX01294 [115, 116].

Existing studies have already identified some H3K9 demethylases, such as JMJD1A/KDM3A, JMJD2A/KDM4A, JMJD2B/KDM4B, JMJD2C/KDM4C, JMJD2D/KDM4D, and the PHD finger proteins 2 and 8 (PHF2 and PHF8) [99, 117]. JMJD1A may regulate the expression of Yes-associated protein 1 (YAP1) and TGF-β2 to increase ECM proteins [118]. Furthermore, JMJD1A reduced H3K9me2 on the CTGF promoter, thereby activating CTGF transcription and promoting myofibroblast activation [119]. Meanwhile, it can also maintain the homeostatic balance of the ECM by binding to the tissue inhibitor matrix metalloproteinase 1 (TIMP1) promoter [120]. KDM4D, another H3K9 demethylase, can inhibit H3K9me2 and H3K9me3, thereby activating the TLR4/MyD88/NF-kB signaling pathway to activate fibroblasts [121]. However, JMJD1A was also discovered to inhibit fibrosis by increasing PPARγ expression [122]. In another study, the sequence-specific transcription factor SREBP2 interacted with KDM4A, B, and C to activate miR-29 transcription, which also plays an anti-fibrotic role [123].

#### H3K4 methylation

MLL family proteins usually act in the histone methyltransferase complex COMPASS, which consists of ASH2, RBBP5, WDR5, and hDPY30 (Fig. 3C, Table 3) [124, 125]. A study found that the components of COMPASS, including WDR5, ASH2, and MLL1, were recruited to the promoters of fibrogenic genes to activate the transcription of collagens in both diabetic nephropathy and CCl4 induced hepatic fibrosis [126, 127]. ASH1 and SET7/9 are two histone methyltransferases that have been found to bind the regulatory regions of ECM genes, TIMP1, CTGF, and TGF-β1, with increased levels of H3K4me1, H3K4me2, and H3K4me3 [128–130].

## DNA methylation

DNA methylation is generally associated with transcriptional silencing. The de novo DNA methylases DNMT3A and DNMT3B can establish a pattern of methylation that is faithfully maintained by the maintenance methyltransferase DNMT1 and associated proteins [131, 132]. In contrast, ten-eleven translocation (TET) proteins convert 5-methylcytosine (5mC) into 5-hydroxymethylcytosine (5hmC), 5-formylcytosine (5fC) and 5-carboxylcytosine (5caC), which can ultimately be removed by thymine-DNA glycosylase (TDG) [133–136]. Therefore the addition and removal of methyl marks is a dynamic process.

CpG islands (CGIs) refer to the CpG-rich regions of DNA, which are often associated with transcription start regions and promoters. Most CGIs remain unmethylated in somatic cells, promoting gene expression, while methylation of CGIs can cause robust transcriptional repression, forming long-term monoallelic silencing, such as X inactivation and genomic imprinting [137, 138]. Transcription factors can be blocked by 5mC directly [139], and then methyl-CpG-binding domain (MBD) proteins bind to the methylated state, leading to indirect repression, which likely requires a high local density of CGs [140]. However, most gene bodies are CpG-poor and are extensively methylated. It has been found that methylation on the gene body may be involved in controlling splicing [137, 141]. In summary, methylation in the promoter region is negatively correlated with the corresponding gene expression, whereas methylation in the gene body is positively correlated with its expression [142]. It is the initiation of transcription but not transcription elongation that seems to be sensitive to DNA methylation silencing [10].

DNA methylation commonly co locates in organ fibrosis (Table 4). To identify specific candidate genes that are hypermethylated in fibrotic fibroblasts, studies have often compared fibroblasts from fibrotic tissues with fibroblasts from nonfibrotic tissues with a genome-wide methylation screen. Three functional categories of genes are stimulated during fibrosis, including cytoskeletal proteins, ECM, and components of the protein synthesis apparatus [143–146].

**Table 4:** Genes promoter hypermethylated/ hypomethylated in organ fibrosis

Organ	Genes
Lung	Thy-1 [154, 155], PTGER2 [158], C8ORF4 [157], TSC1/TSC2 [162], BMPER [163], p14ARF [168], CDKN2B [171]
Heart	Rasal1 [149, 150], COL1A1(hypomethylated) [174, 175], α-SMA(hypomethylated) [174]
Liver	PTEN [152], PPARγ [152, 153], RCAN1.4 [165], SUN2 [166], SEPT9 [167], SMAD7 [169]
Kidney	Rasal1 [143, 147, 148], Klotho [151], SMAD7 [170], sFRP5 [161], KLF4 [172]
Skin	FLI1 [159], sFRP1 [160], SOCS3 [164]

Rasal1 and Klotho are uniquely methylated in renal fibrosis, where Rasal1 and Klotho have been revealed to play a role in fibroblast proliferation and ECM production [143]. Under physiological conditions, the Rasal1 CGI in the promoter region is unmethylated, and Rasal1 is open for transcription. TGF-β1 caused the methylation of CGI, where naked cytosine is transferred to 5mC via the enzyme Dnmt1, causing transcriptional silencing of Rasal1. Further study proved that BMP7 and hydralazine induced Tet3 to convert 5mC into 5hmC and eventually reverted to naked cytosine by TDG, and Rasal1 was reopened for transcription [147, 148]. Furthermore, considering that Tet3 can direct the CXXC motif of CGI within the Rasal1 promoter region, the demethylating activity of BMP and hydralazine is more specific for aberrantly methylated genes [148]. Following these studies, Rasal1 was also found to contribute to EndMT of endothelial cells in cardiac fibrosis [149, 150]. In addition, a study found that Klotho was hypermethylated in renal fibrosis via a similar mechanism [151].

Several genes are highlighted explicitly in liver fibrosis, such as PTEN and PPARγ. PTEN hypermethylation mediated by DNMT1 caused the diminution of PTEN expression, followed by the activation of the PI3K/AKT and ERK pathways, blocking cell proliferation and ECM gene expression in activated HSCs [152]. Described as an antifibrotic gene, the hypermethylation of PPARγ has been studied, and it has been confirmed that in mild liver fibrosis, the PPARγ promoter region is hypomethylated compared with severe fibrosis [153]. On this basis, plasma DNA can be detected and potentially used for noninvasively stratifying fibrosis risk evaluation according to methylation levels at differentially methylated regions (DMRs) within the PPARγ gene promoter region. Thus suggesting cell free DNA could be a possible biomarker in liver fibrosis which would be an important non-invasive method.

In pulmonary fibrosis, the roles of COX-2 and thy-1 in epigenetic regulation have already been established [154–156]. The regulation of COX-2 includes DNA methylation of the COX-2 gene itself and other related genes, such as C8ORF4, a transcriptional regulator of COX-2 in fibrotic lung fibroblasts [157]. Given that COX-2 affects PGE2, it is reasonable to speculate that PGE2 receptors are also involved in the fibrosis process. It was found that the downregulation of PTGER2 and consequent PGE2 resistance were both mediated by DNA hypermethylation [158]. The function of PGE2 in promoting fibroblast proliferation was also related to the increase in the expression of DNMT3a.

Friend leukemia integration 1 (FLI1) has already been identified as an antifibrotic gene hypermethylated in SSc fibroblasts. The FLI1 proximal promoter region can be methylated and bind to MeCP2, which then recruits HDAC to the promoter [159]. MeCP2 which is a protein that binds to methylated DNA thus aiding the transcriptional repression was also found to be overexpressed in SSc fibroblast and skin. Using lentiviral overexpression of MeCP2 in normal skin fibroblasts it was found that this led to myofibroblast formation and collagen expression. Mechanistically MeCP2 bound to the methylated promoter of the Wnt antagonist sFRP1 thus leading to enhanced Wnt signaling leading to fibrosis [160], another gene in this family, sFRP5 was found to promote fibrosis in CKD [161].

In recent years, with the help of technological advances in identifying DNA hypermethylation, other common targets of fibrosis have been detected; for example, hypermethylation in the promoter region of tuberous sclerosis complexes 1 and 2 (TSC1 and TSC2) [162], bone morphogenetic protein-binding endothelial regulator (BMPER) [163], suppressor of cytokine signaling 3 (SOCS3) [164], regulator of calcineurin 1, isoform 4 (RCAN1.4) [165], Sad1 and UNC84 domain containing 2 (SUN2) [166], SEPT9 [167], p14ARF [168], Smad7 [169, 170], CDKN2B [171] and KLF4 [172] are involved in fibroblast activation, apoptosis and EMT, respectively. In contrast, hypermethylation in the gene body of the β1-subunit of the calcium-sensitive potassium channel (KCNMB1) can attenuate α-SMA expression with an increase in potassium ion channel activity [173].

Although most experiments have focused on hypermethylation in the promoter region in fibrosis, global DNA methylation analysis has also found that some profibrotic gene promoters are hypomethylated, resulting in increased gene expression. In these specific studies, the expression of DNMTs is controversial. The reason for the discrepant findings is still unclear but may be related to the different cell types or experimental conditions used [174, 175]. However, CRISPR/Cas9-mediated epigenome editing is a technology of great specificity. It has been used to verify known and to explore unknown DNA hypermethylation genes associated with organ fibrosis in recent years [176, 177]. Recent research generated a high-fidelity CRISPR/Cas9-based gene-specific dioxygenase by fusing an endonuclease-deactivated high-fidelity Cas9 (dHFCas9) to the TET3 catalytic domain (TET3CD), promoting a more specific reactivation of the targeted gene by guiding RNAs. CRISPR/dCas9-mediated epigenome editing was first applied in fibrotic disease to confirm the reversal of DNA methylation by TET3 in Klotho [176]. The functional role of a matrix stiffness-regulated mechanosensitive gene, desmoplakin (DSP), was discovered using this method [177].

## Noncoding RNAs

Noncoding RNAs, which are transcribed from DNA but are not translated into proteins, are related to epigenetics and can be grouped in three categories: short ncRNAs, long ncRNAs and circular ncRNAs.

### MicroRNAs

MicroRNAs (miRNAs) are short noncoding RNAs of ~ 22 nucleotides that mediate gene silencing by guiding argonaute (AGO) proteins to target sites in the 3’ untranslated regions (UTRs) of mRNAs [178]. miRNA-loaded AGO forms the targeting module of the miRNA-induced silencing complex (miRISC), which promotes translation repression and mRNA degradation [179]. Moreover, miRNAs form a complex network of interactions, as one miRNA can silence hundreds of genes and multiple miRNAs can regulate the same gene [180]. However, some unconventional roles of miRNAs have been discovered to activate gene expression in a mechanism that requires further study [181]. It has been shown that microRNAs are closely associated with epigenetics. Epigenetic modifications have been demonstrated to affect miRNA expression, and miRNAs that control the epigenetic machinery by targeting its enzymatic components are called epi-miRNAs [182].

Histone modifications have been revealed to play a role in the downregulation of miR-133a expression in cardiac fibrosis since HDAC1 and HDAC2 are present in the miR-133a enhancer regions [183]. DNA methylation has also been demonstrated to suppress miR-149 and miR-150 expression in the skin fibrosis and liver fibrosis processes, leading to the repression of the targeting genes [184, 185]. Epi-miRNAs have been discovered in fibrotic tissue in different organs. MiR-29a downregulates HDAC4 [186], while miR-489 downregulates HDAC2 [187], resulting in the decreased expression of ECM. In addition, miR-29b and miR-185 target DNMTs, which epigenetically regulate PTEN and MEG3 expression in liver and kidney fibrosis [188, 189]. Interestingly, a novel epigenetic feedback loop was formed between the miR-17 ~ 92 miRNA cluster and DNMT-1 in IPF. MiR-17 ~ 92 expression is reduced in lung fibroblasts due to increased methylation via DNMT1. Several miRNAs from the miR-17 ~ 92 cluster target DNMT-1 expression, resulting in a negative feedback loop [190]. Also, miR132 was found to be dysregulated in SSc and regulated MeCP2 leading to enhanced fibrosis [160].

### LncRNAs

Long noncoding RNAs (lncRNAs) refer to RNA transcripts with a length > 200 nt that do not encode proteins [191]. LncRNAs are often defined by their location relative to nearby protein-coding genes, including antisense lncRNAs, intronic lncRNAs, bidirectional lncRNAs, and intergenic lncRNAs [192]. Currently, the most studied function of lncRNAs is transcription regulation, which includes chromatin modulation, general transcription machinery, and specific transcription factors. However, apart from transcription regulation, lncRNAs also function in posttranscriptional regulation, organization of protein complexes, cell–cell signaling, and allosteric regulation of proteins [193]. Overall, lncRNAs can be summarized to function as signals, decoys, guides, and scaffolds.

Most lncRNAs play roles in fibrosis through miRNAs as ceRNAs in posttranslational regulation, while others regulate chromatin structure, nuclear translocation, and the binding of transcription factors at the transcription level (Additional file 1: Table. S1). In addition, it has been found that some lncRNAs can regulate fibrosis through mRNA processing and help to maintain mRNA stability after transcription.

### CircRNAs

circRNAs are covalently closed through back-splicing, in which the 5’ end is joined to the 3’ end. CircRNAs act through multiple mechanisms, including transcription regulation, miRNA sponge, protein binding and peptide translation, which are similar to lncRNAs [194], yet relatively more resistant to exonucleases than linear RNAs.

Most previous studies were focused on the miRNA sponge mechanism of circRNA to regulate the transcription of pro-fibrotic genes [195–198]. However, some enlightening studies have revealed the novel mechanism of circRNAs in fibrosis. CircSCAR, located in mitochondria of HSCs, binds to ATP5B, shuts down mPTP, increases the output of ROS, and finally induces hepatic fibrosis [199]. CircYAP, binds to tropomyosin-4 (TMP4) and gamma-actin (ACTG), resulting in the inhibition of actin polymerization and the following cardiac fibrosis [200]. CircHECTD1 decrease and HECTD1 increase were discovered in SiO2-induced pulmonary fibrosis, which indicates a pre-mRNA competition mechanism between circRNA and mRNA [201].

## Epigenetic therapies

Epigenetic modifications are reversible, making them good candidates for potential therapeutic targets [202]. All the epigenetic proteins described above can be addressed through small-molecule inhibitors.

HDAC inhibitors are among the most popular epigenetic drugs currently being evaluated. In many preclinical studies, trichostatin A (TSA) [87, 203], valproic acid (VPA) [89], vorinostat (suberoylanilide hydroxamic acid, SAHA) [78], panobinostat (LBH589) [78, 79], and pracinostat (SB939) [85] have been found to be pan-HDAC inhibitors which are potential treatments for fibrosis in IPF, SSc, CKD, NAFLD and MI patients. Four HDACIs, the selective type I HDAC inhibitor mocetinostat [204], the selective HDAC8 inhibitor NCC170 [82], and the HDAC6 inhibitors tubastatin A and tubacin [204], have been approved by the FDA for the treatment of hematological tumors yet none is clinically applied in fibrotic disease [205]. DZNeP and GSK126 [206] are both EZH2 inhibitors that are commonly used in research of different organ fibrosis. JQ1, a BET inhibitor, has been confirmed to treat liver fibrosis in preclinical research [207]. CM-272 has been tested as an inhibitor of both G9a and DNMT1 treating cirrhotic livers [208].

DNMT inhibitors are types of epigenetic therapy that have been under development for a long period of time. The most commonly used interventions in preclinical studies of fibrosis mainly include 5-aza-2'-deoxycytidine and 5-aza [152, 163, 167, 169, 209]. These two famous DNMT inhibitors have already been clinically applied under the names azacitidine and decitabine for many tumors and are generally well tolerated [210]. However, the same problem exists: currently, the most widely used DNMT inhibitors lack specificity.

In recent years, research on miRNA therapies has emerged [211]. Potential strategies include miRNA mimics to simulate miRNA function and antimiRs to inhibit miRNA function [212]. Preclinical research has revealed that miR-21 in cardiac fibroblasts inhibits SPRY1 protein expression, resulting in fibroblast proliferation. The injection of a specific antisense microribonucleic acid against miR-21 can lead to the regression of cardiac fibrosis [213, 214]. Another study also found that treatment with a miR-29b mimic restored the bleomycin-induced reduction in miR-29 and blocked or even reversed pulmonary fibrosis [215]. Moreover, remolarsen, a miR-29b mimic, is under clinical research for keloids (NCT03601052) [216]. However, only a few miRNA therapeutics have advanced into the clinical testing stage. The greatest challenge is to identify the best miRNA candidates or miRNA targets for each specific disease.

However, none of the interventions mentioned above have been applied for any fibrotic disease in clinical trials due to their limitations in two aspects: the uncertainty of the therapeutic effect of epigenetic therapies in fibrotic diseases and possible adverse effects. The preclinical research of epigenetics in fibrotic diseases is relatively immature compared with that in oncology. Studies evaluating the therapeutic effect are far from sufficient. Different results may be found in studies of different fibrosis models, different stages of fibrosis, and different dosages of drugs. Thus, further studies of the complex apparent regulatory network are necessary. Only after fully understanding each pathway can the effectiveness of its therapeutic targets be determined. Another problem involves the adverse effects of epigenetic interventions. The currently available epigenetic therapies do not target a specific gene or cell type, which may induce unexpected results. Therefore, specific delivery systems and CRISPR technology may provide solutions with higher specificity.

## Conclusions

In past decades, studies have revealed the vital role epigenetic regulation plays in organ fibrosis [217]. With the development of new technologies, our understanding of the mechanisms of fibrosis has deepened dramatically, with effects ranging from chromatin changes to gene expression. Different epigenetic regulations are involved in every phase of fibrosis. Transcription is the main target of epigenetic modifications, among which regulation of promoter regions shows the highest importance. Some regulations are achieved through a single epigenetic modification, while it is more often the case that multiple epigenetic modifications participate together to form a complex network. Epigenetic interventions have been evaluated and applied in clinical use. HDAC inhibitors and DNMT inhibitors are the most studied, but there has been no clinical research on fibrotic diseases. We believe that translation from preclinical to clinical research is necessary and even urgent and call for more efforts on this topic. In addition to their therapeutic potential for organ fibrosis, epigenetic factors can also be used as accurate predictive biomarkers for the diagnosis and prognosis of fibrotic diseases. For example, plasma DNA can be detected and potentially used to noninvasively stratify fibrosis risk according to methylation levels at DMRs within the targeted gene promoter. Much more studies analyzing plasma cell free DNA as a biomarker for fibrotic disease are needed.

The epigenomics project is already in full swing. The mapping of detailed human DNA methylomes, histone modification, and nucleosome positioning maps in healthy and diseased tissues facilitates both basic research and the clinical application of epigenetics in fibrotic diseases. Ultimately, the transformation from biological to clinical research will enable epigenetic regulation to achieve greater value in predicting, diagnosing, and treating fibrotic diseases.

## Supplementary Information

**Additional file 1**: **Table S1.** lncRNAs involved in fibrosis of different organs.

## Data Availability

Not applicable.

## Abbreviations

ECM: Extracellular matrix
ncRNA: Noncoding RNA
TGF-β: Transforming growth factor-β
PDGF: Platelet-derived growth factor
TF: Transcription factors
ATP: Adenosine triphosphate
DBP: DNA-binding protein
BRG1: Brahma-related gene 1
EndMT: Endothelial-to-mesenchymal transition
HAT: Histone acetylase
HDAC: Histone deacetylase
GNAT: Non-derepressible 5-related N-acetyltransferases
CCL2: C–C motif chemokine ligand 2
NOX4: NADPH oxidase 4
BRD4: Bromodomain-containing protein 4
SNAI1: Snail family transcriptional repressor 1
CTGF: Connective tissue growth factor
SIRT: Sirtuins
COX2: Cyclooxygenase 2
IP-10: 10 KDa interferon gamma-induced protein
PPARG: Peroxisome proliferator-activated receptor gamma
PAI1: Plasminogen activator inhibitor 1
α-SMA: α-Smooth muscle actin
AFT3: Transcription factor 3
WIF1: WNT inhibitory factor 1
MAP2K3: Mitogen-activated protein kinase kinase 3
Thy1: Thymocyte differentiation antigen 1
PPARGC1A: Peroxisome proliferator-activated receptor γ coactivator 1
MMP9: Matrix metallopeptidase 9
HGF: Hepatocyte growth factor
IGF: Insulin-like growth factor
EZH2: Enhancer of zeste homolog 2
EED: Embryonic ectoderm development
SUZ12: Suppressor of zeste 12
PRC2: Polycomb repressive complex 2
PTEN: Phosphatase and tensin homolog deleted on chromosome 10
JMJD3: Jumonji domain-containing protein D 3
UTX: Ubiquitously transcribed tetratricopeptide repeat on chromosome X
FRA2: Fos-related antigen 2
XIAP: X-linked inhibitor of apoptosis protein
IAP: Inhibitor of apoptosis protein
HP1: Heterochromatin protein 1
PHF2: PHD Finger Protein 2
PHF8: PHD Finger Protein 8
YAP1: Yes-associated protein 1
TIMP1: Tissue inhibitor matrix metalloproteinase 1
TET: Ten-eleven translocation
5mC: 5-Methylcytosine
5hmC: 5-Hydroxymethylcytosine
TDG: Thymine-DNA glycosylase
CGI: CpG island
MBD: Methyl-CpG-binding domain
RASAL1: RAS protein activator like 1
BMP-7: Bone morphogenetic protein 7
DMR: Differentially methylated region
FLI1: Friend leukemia integration 1
dHFCas9: Deactivated high-fidelity Cas9
TET3CD: TET3 catalytic domain
miRNAs: MicroRNAs
AGO: Argonaute
UTR: Untranslated region
miRISC: MiRNA-induced silencing complex
MEG3: Maternally expressed 3
lncRNA: Long noncoding RNAs
