# Supplementary material for: Epigenetics as a versatile regulator of fibrosis
Source: J Transl Med. 2023 Mar 2;21:164. doi: 10.1186/s12967-023-04018-5 (PMC9983257; doi:10.1186/s12967-023-04018-5)
Supplement: Supplementary file 1 — Additional file 1: Table S1. lncRNAs involved in fibrosis of different organs. [file 12967_2023_4018_MOESM1_ESM.docx]

**Table S1.** lncRNAs involved in fibrosis of different organs

| **lncRNA** | **organ** | **function** | | **signaling pathway** | **location** | **mechanism** | **ref** | |
| --- | --- | --- | --- | --- | --- | --- | --- | --- |
| MALAT1 | liver | pro | HSC proliferation | MALAT1-miR101b-Rac1 | cytoplasma | ceRNA(post-transcription) | [1] |  |
| PVT1 | liver | pro | EMT | PVT1-miR-152-PTCH1(Hedgehog signalling) | cytoplasma | ceRNA(post-transcription) | [2] |  |
| NEAT1 | liver | pro | HSC activation and autophagy | 1. NEAT1-miR-122-KLF6（TGFβ signalling）;  2. NEAT1-miR-29b/Atg9a | cytoplasma | ceRNA(post-transcription) | [3, 4] |  |
| LFAR1 | liver | pro | HSC activation | 1. LFAR1-Smad2/3+TGFβR1-Smad2/3 phosphorylation-Smd2/3 nuclear translocation-profibrotic gene expression(TGFβ/ Smad signalling);  2. LFAR1+Smad2/3-Notch related gene expression | cytoplasma+nucleus | transcription factor(transcription) | [5] |  |
| ATB | liver | pro | HSC activation | 1. ATB-miR-425-5p-TGFβR2/SMAD2;  2. ATB-miR-200a-β-catenin(Wnt signalling) | cytoplasma | ceRNA(post-transcription) | [6, 7] |  |
| HOTAIR | liver | pro | HSC activation | 1. HOTAIR-miR-29b-DNMT3b-PTEN(ERK and AKT signalling);  2. HOTAIR-miR-148b-DNMT1-MEG3;3. HOTAIR-PCR2 recruiment-MEG3 H3K27me3 | cytoplasma+nucleus | ceRNA(post-transcription);  chromatin modulation(transcription) | [8] |  |
| H19 | liver | pro | EMT | H19+ZEB1-EpCAM | nucleus | transcription factor(transcription) | [9] |  |
| SCARNA10 | liver | pro | HSC activation and HC apoptosis | SCARNA10+PCR2-profibrotic gene expression | nucleus | transcription regulator(transcription) | [10] |  |
| linc-SCRG1 | liver | pro | HSC activation | linc-SCRG1+TTP-MMP-2 and TNF-α mRNA stability | cytoplasma | mRNA stability control(post-transcription) | [11] |  |
| linc-p21 | liver | pro | HSC proliferation and apoptosis | 1. linc-p21-miR-30-KLF11; | cytoplasma | ceRNA(post-transcription) | [12] |  |
| HOTTIP | liver | pro | HSC activation | 1.HOTTIP-miR-150-SRF;  2. HOTTIP-miR-148a-TGFBR1 and TGFBR2 | cytoplasma | ceRNA(post-transcription) | [13, 14] |  |
| SNHG7 | liver | pro | HSC activation | SNHG7-miR-378a-3p-DVL2(Wnt signalling) | cytoplasma | ceRNA(post-transcription) | [15] |  |
| linc-p21 | liver | anti | HSC activation | 1. linc-p21-MicroRNA-17-5p-β-catenin(Wnt signalling);  2. linc-p21-miR-181b-PTEN | cytoplasma | ceRNA(post-transcription) | [16] |  |
| MEG3 | liver | anti | EMT | 1. MEG3+SMO-↓GLI3 nuclear translocation(Hedgehog signalling);  2. MEG3-miR-212-Ptch1(Hedgehog signalling) | cytoplasma | transcription factor(transcription);  ceRNA(post-transcription) | [17] |  |
| GAS5 | liver | anti | HSC activation | 1. GAS5-miR-222-p27;  2. miR-23a-PTEN(Akt signalling) | cytoplasma | ceRNA(post-transcription) | [18, 19] |  |
| H19 | liver | anti | HSC activation | H19-ERK1/2 nuclear translocation(ERK signaling) | nucleus | transcription factor(transcription) | [20] |  |
| H19 | heart | pro | ECM production | H19-miR-455-CTGF | cytoplasma | ceRNA(post-transcription) | [21] |  |
| Wisper | heart | pro | ECM production | Wisper+TIAR-PLOD2 | cytoplasma+nucleus | mRNA processing | [22] |  |
| MEG3 | heart | pro | ECM deposition | MEG3-p53 nuclear translocation-MMP2 expression | cytoplasma | transcription factor(transcription) | [23] |  |
| MIAT | heart | pro | fibroblast activation | MIAT-miR-24-Furin and TGFb1 | cytoplasma | ceRNA(post-transcription) | [24] |  |
| Safe | heart | pro | fibroblast activation | Safe-HuR recruiment-Sfrp2 mRNA stability | cytoplasma | mRNA stability control(post-transcription) | [25] |  |
| PFL | heart | pro | fibroblast activation | PFL-let-7d-Ptafr | cytoplasma | ceRNA(post-transcription) | [26] |  |
| MALAT1 | heart | pro | fibroblast activation | MALAT1-miR-145/miR-141-TGF-β signaling | cytoplasma | ceRNA(post-transcription) | [27, 28] |  |
| PCFL | heart | pro | ECM production | PCFL-miR-378-GRB2 | cytoplasma | ceRNA(post-transcription) | [29] |  |
| SRA1 | heart | pro | fibroblast activation | SRA1-miR-148b | cytoplasma | ceRNA(post-transcription) | [30] |  |
| RNF7 | heart | pro | fibroblast activation | RNF7-miR-543-TSP1(TGFb signaling) | cytoplasma | ceRNA(post-transcription) | [31] |  |
| SNHG7 | heart | pro | fibroblast activation | SNHG7-miR-34-5p-ROCK1 | cytoplasma | ceRNA(post-transcription) | [32] |  |
| GAS5 | heart | anti | fibroblast activation | GAS5miR-21-PTEN-MMP-2 | cytoplasma | ceRNA(post-transcription) | [33] |  |
| Linc00092 | heart | anti | fibroblast activation | Linc00092-ERK dephosphate | cytoplasma | transcription factor(transcription) | [34] |  |
| Erbb4-IR | kidney | pro | fibroblast activation | 1. Erbb4-IR+smad7 genomic sequence-↓smad7;  2. Erbb4-IR-miR-29-collagen I and collagen IV | nucleus+cytoplasma | lncRNA-DNA triplex structures(transcription);  ceRNA(post-transcription); | [35, 36] |  |
| H19 | kidney | pro | ECM synthesis | lncRNA-H19-miR-17-fibronectin | cytoplasma | ceRNA(post-transcription) | [37] |  |
| MALAT1 | kidney | pro | EMT and ECM production | 1. MALAT1-miR-145/ZEB2/FAK;  2. MALAT1+SRSF1-β-catenin mRNA splicing | cytoplasma+nucleus | ceRNA(post-transcription);  mRNA processing | [38-40] |  |
| NR_033515 | kidney | pro | EMT and ECM production | NR_033515-miR-743b-5p-profibrotic gene expression | cytoplasma | ceRNA(post-transcription) | [41] |  |
| MEG3 | kidney | pro | ECM production | MEG3-miR-181a-Egr-1 | cytoplasma | ceRNA(post-transcription) | [42] |  |
| HOTAIR | kidney | pro | EMT and ECM production | HOTAIR-miR-124-Notch1(Notch signaling) | cytoplasma | ceRNA(post-transcription) | [43, 44] |  |
| MIAT | kidney | pro | EMT and ECM production | MIAT-miR-145-EIF5A2;MIAT-miR-147a-E2F3 | cytoplasma | ceRNA(post-transcription) | [45, 46] |  |
| LOC105375913 | kidney | pro | ECM production | LOC105375913-miR-27b-snail | cytoplasma | ceRNA(post-transcription) | [47] |  |
| TSI | kidney | anti | fibroblast activation | TSI+Smad3-↓Smad3 phosphorylation-↓Smad3 nuclear translocation-↓profibrotic gene expression(TGFβ/Smad signalling) | cytoplasma | transcription factor(transcription) | [48] |  |
| Linc-1700020I14Rik | kidney | anti | fibroblast activation | Linc-1700020I14Rik-miR-34a-5p-Sirt1-HIF-1α | cytoplasma | ceRNA(post-transcription) | [49] |  |
| GAS5 | kidney | anti | ECM production and ECM deposition | 1. GAS5-miR-221-SIRT1-?;  2. GAS5-EZH2 recruiment-MMP9 H3K27me3 | cytoplasma+nucleus | ceRNA(post-transcription);  chromatin modulation(transcription) | [50, 51] |  |
| NR_038323 | kidney | anti | ECM production | NR_038323-miR-324-3p-DUSP1(MAPK signaling) | cytoplasma | ceRNA(post-transcription) | [52] |  |
| ZEB1-AS1 | kidney | anti | ECM production | lncRNA ZEB1-AS1-MLL1 recruiment-ZEB1 H3K4me3 | nucleus | chromatin modulation(transcription) | [53] |  |
| XIST | kidney | anti | ECM production | XIST-miR-93-5p-CDKN1A | cytoplasma | ceRNA(post-transcription) | [54] |  |
| PVT1 | kidney | anti | ECM production | PVT1-miR-181a-5p-TGF-βR1 | cytoplasma | ceRNA(post-transcription) | [55] |  |
| MRAK088388/MRAK081523 | lung | pro | fibroblast proliferation | 1. MRAK088388-miR-29b-3p-N4bp2;  2. MRAK081523-let-7i-5p-Plxna4 | cytoplasma | ceRNA(post-transcription) | [56] |  |
| PCF | lung | pro | myofibroblast proliferation | PCF-miR-344a-5p-map3k11 | cytoplasma | ceRNA(post-transcription) | [57] |  |
| H19 | lung | pro | EMT and fibroblast activation | 1. H19-miR-29b-COL1A1 and Acta2;  2. H19-miR-196a-COL1A1;  3. H19-miR-140-TGFb signaling | cytoplasma | ceRNA(post-transcription) | [58-60] |  |
| CHRF | lung | pro | fibroblast activation | CHRF-miR-489-Smad3(TGF-β signaling) | cytoplasma | ceRNA(post-transcription) | [61] |  |
| MALAT1 | lung | pro | EMT | MALAT1-MiR-503-PI3K/Akt/mTOR/Snail | cytoplasma | ceRNA(post-transcription) | [62] |  |
| ITPF | lung | pro | fibroblast activation | lncITPF+hnRNP-L-ITGBL1 | nucleus | mRNA processing | [63] |  |
| ATB | lung | pro | EMT | ATB-miR-200c-ZEB1 | cytoplasma | ceRNA(post-transcription) | [64] |  |
| PFAR | lung | pro | ECM deposition | miR-138-YAP1 | cytoplasma | ceRNA(post-transcription) | [65] |  |
| ncm3os | lung | pro | fibroblast activation | ncm3os-miR-199a-5p/CAV1,miR-199a-3p/FGF7 and HGF,miR-214-3p/GSK-3β/β-catenin | cytoplasma | Reservoir of miRs | [66, 67] |  |
| PFRL | lung | pro | fibroblast activation | PFRL-miR-26a-smad2 | cytoplasma | ceRNA(post-transcription) | [68] |  |
| Hoxaas3 | lung | pro | fibroblast activation | Hoxaas3-miR-450b-5p-Runx1 | cytoplasma | ceRNA(post-transcription) | [69] |  |
| DANCR | lung | pro | EMT and ECM production | DANCR+AUF1-FOXO3 mRNA | cytoplasma | mRNA stability control(post-transcription) | [70] |  |
| NEAT1 | lung | pro | EMT | NEAT1-miR-9-5p-TGF-β1/Smad2 | cytoplasma | ceRNA(post-transcription) | [71] |  |
| FENDRR | lung | anti | fibroblast activation | FENDRR-microRNA-214- | cytoplasma | ceRNA(post-transcription) | [72] |  |
| sirt1 AS | lung | anti | EMT | sirt1 AS- sirt mRNA stability-Akt/foxo3 | cytoplasma | mRNA stability control(post-transcription) | [73] |  |
| HOXA11-AS | skin | pro | fibroblast activation | HOXA11-AS-miR-124-3p-Smad5/TGFβR1 | cytoplasma | ceRNA(post-transcription) | [74, 75] |  |
| HOTAIR | skin | pro | fibroblast activation | HOTAIR-EZH2 recruiment-miR34a H3K27me3-notch1 | nucleus | chromatin modulation(transcription) | [76] |  |
| ATB | skin | pro | fibroblast activation | ATB -miR-200c-ZNF217(TGF-β signaling) | cytoplasma | ceRNA(post-transcription) | [77] |  |
| H19 | skin | pro | fibroblast activation | H19-miR-29a-COL1A1 | cytoplasma | ceRNA(post-transcription) | [78] |  |
| GAS5 | skin | anti | fibroblast activation | GAS5-Smad3+PPM1A-Smad3 dephosphatase | cytoplasma | transcription factor(transcription) | [79] |  |

Reference

1. Yu F, Lu Z, Cai J, Huang K, Chen B, Li G, Dong P, Zheng J: **MALAT1 functions as a competing endogenous RNA to mediate Rac1 expression by sequestering miR-101b in liver fibrosis.** *Cell Cycle* 2015, **14:**3885-3896.

2. Zheng J, Yu F, Dong P, Wu L, Zhang Y, Hu Y, Zheng L: **Long non-coding RNA PVT1 activates hepatic stellate cells through competitively binding microRNA-152.** *Oncotarget* 2016, **7:**62886-62897.

3. Yu F, Jiang Z, Chen B, Dong P, Zheng J: **NEAT1 accelerates the progression of liver fibrosis via regulation of microRNA-122 and Kruppel-like factor 6.** *J Mol Med (Berl)* 2017, **95:**1191-1202.

4. Kong Y, Huang T, Zhang H, Zhang Q, Ren J, Guo X, Fan H, Liu L: **The lncRNA NEAT1/miR-29b/Atg9a axis regulates IGFBPrP1-induced autophagy and activation of mouse hepatic stellate cells.** *Life Sci* 2019, **237:**116902.

5. Zhang K, Han X, Zhang Z, Zheng L, Hu Z, Yao Q, Cui H, Shu G, Si M, Li C, et al: **The liver-enriched lnc-LFAR1 promotes liver fibrosis by activating TGFβ and Notch pathways.** *Nat Commun* 2017, **8:**144.

6. Fu N, Niu X, Wang Y, Du H, Wang B, Du J, Li Y, Wang R, Zhang Y, Zhao S, et al: **Role of LncRNA-activated by transforming growth factor beta in the progression of hepatitis C virus-related liver fibrosis.** *Discov Med* 2016, **22:**29-42.

7. Fu N, Zhao SX, Kong LB, Du JH, Ren WG, Han F, Zhang QS, Li WC, Cui P, Wang RQ, et al: **LncRNA-ATB/microRNA-200a/β-catenin regulatory axis involved in the progression of HCV-related hepatic fibrosis.** *Gene* 2017, **618:**1-7.

8. Yu F, Chen B, Dong P, Zheng J: **HOTAIR Epigenetically Modulates PTEN Expression via MicroRNA-29b: A Novel Mechanism in Regulation of Liver Fibrosis.** *Mol Ther* 2020, **28:**2703.

9. Song Y, Liu C, Liu X, Trottier J, Beaudoin M, Zhang L, Pope C, Peng G, Barbier O, Zhong X, et al: **H19 promotes cholestatic liver fibrosis by preventing ZEB1-mediated inhibition of epithelial cell adhesion molecule.** *Hepatology* 2017, **66:**1183-1196.

10. Zhang K, Han Y, Hu Z, Zhang Z, Shao S, Yao Q, Zheng L, Wang J, Han X, Zhang Y, et al: **SCARNA10, a nuclear-retained long non-coding RNA, promotes liver fibrosis and serves as a potential biomarker.** *Theranostics* 2019, **9:**3622-3638.

11. Wu JC, Luo SZ, Liu T, Lu LG, Xu MY: **linc-SCRG1 accelerates liver fibrosis by decreasing RNA-binding protein tristetraprolin.** *Faseb j* 2019, **33:**2105-2115.

12. Tu X, Zhang Y, Zheng X, Deng J, Li H, Kang Z, Cao Z, Huang Z, Ding Z, Dong L, et al: **TGF-β-induced hepatocyte lincRNA-p21 contributes to liver fibrosis in mice.** *Sci Rep* 2017, **7:**2957.

13. Zheng J, Mao Y, Dong P, Huang Z, Yu F: **Long noncoding RNA HOTTIP mediates SRF expression through sponging miR-150 in hepatic stellate cells.** *J Cell Mol Med* 2019, **23:**1572-1580.

14. Li Z, Wang J, Zeng Q, Hu C, Zhang J, Wang H, Yan J, Li H, Yu Z: **Long Noncoding RNA HOTTIP Promotes Mouse Hepatic Stellate Cell Activation via Downregulating miR-148a.** *Cell Physiol Biochem* 2018, **51:**2814-2828.

15. Yu F, Dong P, Mao Y, Zhao B, Huang Z, Zheng J: **Loss of lncRNA-SNHG7 Promotes the Suppression of Hepatic Stellate Cell Activation via miR-378a-3p and DVL2.** *Mol Ther Nucleic Acids* 2019, **17:**235-244.

16. Yu F, Guo Y, Chen B, Shi L, Dong P, Zhou M, Zheng J: **LincRNA-p21 Inhibits the Wnt/β-Catenin Pathway in Activated Hepatic Stellate Cells via Sponging MicroRNA-17-5p.** *Cell Physiol Biochem* 2017, **41:**1970-1980.

17. Yu F, Geng W, Dong P, Huang Z, Zheng J: **LncRNA-MEG3 inhibits activation of hepatic stellate cells through SMO protein and miR-212.** *Cell Death Dis* 2018, **9:**1014.

18. Yu F, Zheng J, Mao Y, Dong P, Lu Z, Li G, Guo C, Liu Z, Fan X: **Long Non-coding RNA Growth Arrest-specific Transcript 5 (GAS5) Inhibits Liver Fibrogenesis through a Mechanism of Competing Endogenous RNA.** *J Biol Chem* 2015, **290:**28286-28298.

19. Dong Z, Li S, Wang X, Si L, Ma R, Bao L, Bo A: **lncRNA GAS5 restrains CCl(4)-induced hepatic fibrosis by targeting miR-23a through the PTEN/PI3K/Akt signaling pathway.** *Am J Physiol Gastrointest Liver Physiol* 2019, **316:**G539-g550.

20. Yang JJ, She Q, Yang Y, Tao H, Li J: **DNMT1 controls LncRNA H19/ERK signal pathway in hepatic stellate cell activation and fibrosis.** *Toxicol Lett* 2018, **295:**325-334.

21. Huang ZW, Tian LH, Yang B, Guo RM: **Long Noncoding RNA H19 Acts as a Competing Endogenous RNA to Mediate CTGF Expression by Sponging miR-455 in Cardiac Fibrosis.** *DNA Cell Biol* 2017, **36:**759-766.

22. Micheletti R, Plaisance I, Abraham BJ, Sarre A, Ting CC, Alexanian M, Maric D, Maison D, Nemir M, Young RA, et al: **The long noncoding RNA Wisper controls cardiac fibrosis and remodeling.** *Sci Transl Med* 2017, **9**.

23. Piccoli MT, Gupta SK, Viereck J, Foinquinos A, Samolovac S, Kramer FL, Garg A, Remke J, Zimmer K, Batkai S, Thum T: **Inhibition of the Cardiac Fibroblast-Enriched lncRNA Meg3 Prevents Cardiac Fibrosis and Diastolic Dysfunction.** *Circ Res* 2017, **121:**575-583.

24. Qu X, Du Y, Shu Y, Gao M, Sun F, Luo S, Yang T, Zhan L, Yuan Y, Chu W, et al: **MIAT Is a Pro-fibrotic Long Non-coding RNA Governing Cardiac Fibrosis in Post-infarct Myocardium.** *Sci Rep* 2017, **7:**42657.

25. Hao K, Lei W, Wu H, Wu J, Yang Z, Yan S, Lu XA, Li J, Xia X, Han X, et al: **LncRNA-Safe contributes to cardiac fibrosis through Safe-Sfrp2-HuR complex in mouse myocardial infarction.** *Theranostics* 2019, **9:**7282-7297.

26. Liang H, Pan Z, Zhao X, Liu L, Sun J, Su X, Xu C, Zhou Y, Zhao D, Xu B, et al: **LncRNA PFL contributes to cardiac fibrosis by acting as a competing endogenous RNA of let-7d.** *Theranostics* 2018, **8:**1180-1194.

27. Huang S, Zhang L, Song J, Wang Z, Huang X, Guo Z, Chen F, Zhao X: **Long noncoding RNA MALAT1 mediates cardiac fibrosis in experimental postinfarct myocardium mice model.** *J Cell Physiol* 2019, **234:**2997-3006.

28. Che H, Wang Y, Li H, Li Y, Sahil A, Lv J, Liu Y, Yang Z, Dong R, Xue H, Wang L: **Melatonin alleviates cardiac fibrosis via inhibiting lncRNA MALAT1/miR-141-mediated NLRP3 inflammasome and TGF-β1/Smads signaling in diabetic cardiomyopathy.** *Faseb j* 2020, **34:**5282-5298.

29. Sun F, Zhuang Y, Zhu H, Wu H, Li D, Zhan L, Yang W, Yuan Y, Xie Y, Yang S, et al: **LncRNA PCFL promotes cardiac fibrosis via miR-378/GRB2 pathway following myocardial infarction.** *J Mol Cell Cardiol* 2019, **133:**188-198.

30. Zhang S, Gao S, Wang Y, Jin P, Lu F: **lncRNA SRA1 Promotes the Activation of Cardiac Myofibroblasts Through Negative Regulation of miR-148b.** *DNA Cell Biol* 2019, **38:**385-394.

31. Ouyang F, Liu X, Liu G, Qiu H, He Y, Hu H, Jiang P: **Long non-coding RNA RNF7 promotes the cardiac fibrosis in rat model via miR-543/THBS1 axis and TGFβ1 activation.** *Aging (Albany NY)* 2020, **12:**996-1010.

32. Wang J, Zhang S, Li X, Gong M: **LncRNA SNHG7 promotes cardiac remodeling by upregulating ROCK1 via sponging miR-34-5p.** *Aging (Albany NY)* 2020, **12:**10441-10456.

33. Tao H, Zhang JG, Qin RH, Dai C, Shi P, Yang JJ, Deng ZY, Shi KH: **LncRNA GAS5 controls cardiac fibroblast activation and fibrosis by targeting miR-21 via PTEN/MMP-2 signaling pathway.** *Toxicology* 2017, **386:**11-18.

34. Chen ZT, Zhang HF, Wang M, Wang SH, Wen ZZ, Gao QY, Wu MX, Liu WH, Xie Y, Mai JT, et al: **Long non-coding RNA Linc00092 inhibits cardiac fibroblast activation by altering glycolysis in an ERK-dependent manner.** *Cell Signal* 2020, **74:**109708.

35. Feng M, Tang PM, Huang XR, Sun SF, You YK, Xiao J, Lv LL, Xu AP, Lan HY: **TGF-β Mediates Renal Fibrosis via the Smad3-Erbb4-IR Long Noncoding RNA Axis.** *Mol Ther* 2018, **26:**148-161.

36. Sun SF, Tang PMK, Feng M, Xiao J, Huang XR, Li P, Ma RCW, Lan HY: **Novel lncRNA Erbb4-IR Promotes Diabetic Kidney Injury in db/db Mice by Targeting miR-29b.** *Diabetes* 2018, **67:**731-744.

37. Xie H, Xue JD, Chao F, Jin YF, Fu Q: **Long non-coding RNA-H19 antagonism protects against renal fibrosis.** *Oncotarget* 2016, **7:**51473-51481.

38. Liu B, Qiang L, Wang GD, Duan Q, Liu J: **LncRNA MALAT1 facilities high glucose induced endothelial to mesenchymal transition and fibrosis via targeting miR-145/ZEB2 axis.** *Eur Rev Med Pharmacol Sci* 2019, **23:**3478-3486.

39. Liu P, Zhang B, Chen Z, He Y, Du Y, Liu Y, Chen X: **m(6)A-induced lncRNA MALAT1 aggravates renal fibrogenesis in obstructive nephropathy through the miR-145/FAK pathway.** *Aging (Albany NY)* 2020, **12:**5280-5299.

40. Hu M, Wang R, Li X, Fan M, Lin J, Zhen J, Chen L, Lv Z: **LncRNA MALAT1 is dysregulated in diabetic nephropathy and involved in high glucose-induced podocyte injury via its interplay with β-catenin.** *J Cell Mol Med* 2017, **21:**2732-2747.

41. Gao J, Wang W, Wang F, Guo C: **LncRNA-NR_033515 promotes proliferation, fibrogenesis and epithelial-to-mesenchymal transition by targeting miR-743b-5p in diabetic nephropathy.** *Biomed Pharmacother* 2018, **106:**543-552.

42. Zha F, Qu X, Tang B, Li J, Wang Y, Zheng P, Ji T, Zhu C, Bai S: **Long non-coding RNA MEG3 promotes fibrosis and inflammatory response in diabetic nephropathy via miR-181a/Egr-1/TLR4 axis.** *Aging (Albany NY)* 2019, **11:**3716-3730.

43. Zhou H, Gao L, Yu ZH, Hong SJ, Zhang ZW, Qiu ZZ: **LncRNA HOTAIR promotes renal interstitial fibrosis by regulating Notch1 pathway via the modulation of miR-124.** *Nephrology (Carlton)* 2019, **24:**472-480.

44. Zhou H, Qiu ZZ, Yu ZH, Gao L, He JM, Zhang ZW, Zheng J: **Paeonol reverses promoting effect of the HOTAIR/miR-124/Notch1 axis on renal interstitial fibrosis in a rat model.** *J Cell Physiol* 2019, **234:**14351-14363.

45. Wang Z, Zhang B, Chen Z, He Y, Ru F, Liu P, Chen X: **The long noncoding RNA myocardial infarction-associated transcript modulates the epithelial-mesenchymal transition in renal interstitial fibrosis.** *Life Sci* 2020, **241:**117187.

46. Ji TT, Qi YH, Li XY, Tang B, Wang YK, Zheng PX, Li W, Qu X, Feng L, Bai SJ: **Loss of lncRNA MIAT ameliorates proliferation and fibrosis of diabetic nephropathy through reducing E2F3 expression.** *J Cell Mol Med* 2020, **24:**13314-13323.

47. Han R, Hu S, Qin W, Shi J, Zeng C, Bao H, Liu Z: **Upregulated long noncoding RNA LOC105375913 induces tubulointerstitial fibrosis in focal segmental glomerulosclerosis.** *Sci Rep* 2019, **9:**716.

48. Wang P, Luo ML, Song E, Zhou Z, Ma T, Wang J, Jia N, Wang G, Nie S, Liu Y, Hou F: **Long noncoding RNA lnc-TSI inhibits renal fibrogenesis by negatively regulating the TGF-β/Smad3 pathway.** *Sci Transl Med* 2018, **10**.

49. Li A, Peng R, Sun Y, Liu H, Peng H, Zhang Z: **LincRNA 1700020I14Rik alleviates cell proliferation and fibrosis in diabetic nephropathy via miR-34a-5p/Sirt1/HIF-1α signaling.** *Cell Death Dis* 2018, **9:**461.

50. Ge X, Xu B, Xu W, Xia L, Xu Z, Shen L, Peng W, Huang S: **Long noncoding RNA GAS5 inhibits cell proliferation and fibrosis in diabetic nephropathy by sponging miR-221 and modulating SIRT1 expression.** *Aging (Albany NY)* 2019, **11:**8745-8759.

51. Zhang L, Zhao S, Zhu Y: **Long noncoding RNA growth arrest-specific transcript 5 alleviates renal fibrosis in diabetic nephropathy by downregulating matrix metalloproteinase 9 through recruitment of enhancer of zeste homolog 2.** *Faseb j* 2020, **34:**2703-2714.

52. Ge Y, Wang J, Wu D, Zhou Y, Qiu S, Chen J, Zhu X, Xiang X, Li H, Zhang D: **lncRNA NR_038323 Suppresses Renal Fibrosis in Diabetic Nephropathy by Targeting the miR-324-3p/DUSP1 Axis.** *Mol Ther Nucleic Acids* 2019, **17:**741-753.

53. Wang J, Pan J, Li H, Long J, Fang F, Chen J, Zhu X, Xiang X, Zhang D: **lncRNA ZEB1-AS1 Was Suppressed by p53 for Renal Fibrosis in Diabetic Nephropathy.** *Mol Ther Nucleic Acids* 2018, **12:**741-750.

54. Yang J, Shen Y, Yang X, Long Y, Chen S, Lin X, Dong R, Yuan J: **Silencing of long noncoding RNA XIST protects against renal interstitial fibrosis in diabetic nephropathy via microRNA-93-5p-mediated inhibition of CDKN1A.** *Am J Physiol Renal Physiol* 2019, **317:**F1350-f1358.

55. Cao L, Qin P, Zhang J, Qiao H, Shi P, Huo H: **LncRNA PVT1 Suppresses the Progression of Renal Fibrosis via Inactivation of TGF-β Signaling Pathway.** *Drug Des Devel Ther* 2020, **14:**3547-3557.

56. Song X, Cao G, Jing L, Lin S, Wang X, Zhang J, Wang M, Liu W, Lv C: **Analysing the relationship between lncRNA and protein-coding gene and the role of lncRNA as ceRNA in pulmonary fibrosis.** *J Cell Mol Med* 2014, **18:**991-1003.

57. Liu H, Wang B, Zhang J, Zhang S, Wang Y, Zhang J, Lv C, Song X: **A novel lnc-PCF promotes the proliferation of TGF-β1-activated epithelial cells by targeting miR-344a-5p to regulate map3k11 in pulmonary fibrosis.** *Cell Death Dis* 2017, **8:**e3137.

58. Tang Y, He R, An J, Deng P, Huang L, Yang W: **The effect of H19-miR-29b interaction on bleomycin-induced mouse model of idiopathic pulmonary fibrosis.** *Biochem Biophys Res Commun* 2016, **479:**417-423.

59. Lu Q, Guo Z, Xie W, Jin W, Zhu D, Chen S, Ren T: **The lncRNA H19 Mediates Pulmonary Fibrosis by Regulating the miR-196a/COL1A1 Axis.** *Inflammation* 2018, **41:**896-903.

60. Wang X, Cheng Z, Dai L, Jiang T, Jia L, Jing X, An L, Wang H, Liu M: **Knockdown of Long Noncoding RNA H19 Represses the Progress of Pulmonary Fibrosis through the Transforming Growth Factor β/Smad3 Pathway by Regulating MicroRNA 140.** *Mol Cell Biol* 2019, **39**.

61. Wu Q, Han L, Yan W, Ji X, Han R, Yang J, Yuan J, Ni C: **miR-489 inhibits silica-induced pulmonary fibrosis by targeting MyD88 and Smad3 and is negatively regulated by lncRNA CHRF.** *Sci Rep* 2016, **6:**30921.

62. Yan W, Wu Q, Yao W, Li Y, Liu Y, Yuan J, Han R, Yang J, Ji X, Ni C: **MiR-503 modulates epithelial-mesenchymal transition in silica-induced pulmonary fibrosis by targeting PI3K p85 and is sponged by lncRNA MALAT1.** *Sci Rep* 2017, **7:**11313.

63. Song X, Xu P, Meng C, Song C, Blackwell TS, Li R, Li H, Zhang J, Lv C: **lncITPF Promotes Pulmonary Fibrosis by Targeting hnRNP-L Depending on Its Host Gene ITGBL1.** *Mol Ther* 2019, **27:**380-393.

64. Liu Y, Li Y, Xu Q, Yao W, Wu Q, Yuan J, Yan W, Xu T, Ji X, Ni C: **Long non-coding RNA-ATB promotes EMT during silica-induced pulmonary fibrosis by competitively binding miR-200c.** *Biochim Biophys Acta Mol Basis Dis* 2018, **1864:**420-431.

65. Zhao X, Sun J, Chen Y, Su W, Shan H, Li Y, Wang Y, Zheng N, Shan H, Liang H: **lncRNA PFAR Promotes Lung Fibroblast Activation and Fibrosis by Targeting miR-138 to Regulate the YAP1-Twist Axis.** *Mol Ther* 2018, **26:**2206-2217.

66. Savary G, Dewaeles E, Diazzi S, Buscot M, Nottet N, Fassy J, Courcot E, Henaoui IS, Lemaire J, Martis N, et al: **The Long Noncoding RNA DNM3OS Is a Reservoir of FibromiRs with Major Functions in Lung Fibroblast Response to TGF-β and Pulmonary Fibrosis.** *Am J Respir Crit Care Med* 2019, **200:**184-198.

67. Jiang D, Liang J: **A Long Noncoding RNA links TGF-β Signaling in Lung Fibrosis.** *Am J Respir Crit Care Med* 2019, **200:**123-125.

68. Jiang H, Chen Y, Yu T, Zhao X, Shan H, Sun J, Zhang L, Li X, Shan H, Liang H: **Inhibition of lncRNA PFRL prevents pulmonary fibrosis by disrupting the miR-26a/smad2 loop.** *Am J Physiol Lung Cell Mol Physiol* 2018, **315:**L563-l575.

69. Lin S, Zhang R, Xu L, Ma R, Xu L, Zhu L, Hu J, An X: **LncRNA Hoxaas3 promotes lung fibroblast activation and fibrosis by targeting miR-450b-5p to regulate Runx1.** *Cell Death Dis* 2020, **11:**706.

70. Qian W, Cai X, Qian Q, Wang D, Zhang L: **Angelica Sinensis Polysaccharide Suppresses Epithelial-Mesenchymal Transition and Pulmonary Fibrosis via a DANCR/AUF-1/FOXO3 Regulatory Axis.** *Aging Dis* 2020, **11:**17-30.

71. Zhang Y, Yao XH, Wu Y, Cao GK, Han D: **LncRNA NEAT1 regulates pulmonary fibrosis through miR-9-5p and TGF-β signaling pathway.** *Eur Rev Med Pharmacol Sci* 2020, **24:**8483-8492.

72. Huang C, Liang Y, Zeng X, Yang X, Xu D, Gou X, Sathiaseelan R, Senavirathna LK, Wang P, Liu L: **Long Noncoding RNA FENDRR Exhibits Antifibrotic Activity in Pulmonary Fibrosis.** *Am J Respir Cell Mol Biol* 2020, **62:**440-453.

73. Qian W, Cai X, Qian Q: **Sirt1 antisense long non-coding RNA attenuates pulmonary fibrosis through sirt1-mediated epithelial-mesenchymal transition.** *Aging (Albany NY)* 2020, **12:**4322-4336.

74. Jin J, Zhai HF, Jia ZH, Luo XH: **Long non-coding RNA HOXA11-AS induces type I collagen synthesis to stimulate keloid formation via sponging miR-124-3p and activation of Smad5 signaling.** *Am J Physiol Cell Physiol* 2019, **317:**C1001-c1010.

75. Jin J, Jia ZH, Luo XH, Zhai HF: **Long non-coding RNA HOXA11-AS accelerates the progression of keloid formation via miR-124-3p/TGFβR1 axis.** *Cell Cycle* 2020, **19:**218-232.

76. Wasson CW, Abignano G, Hermes H, Malaab M, Ross RL, Jimenez SA, Chang HY, Feghali-Bostwick CA, Del Galdo F: **Long non-coding RNA HOTAIR drives EZH2-dependent myofibroblast activation in systemic sclerosis through miRNA 34a-dependent activation of NOTCH.** *Ann Rheum Dis* 2020, **79:**507-517.

77. Zhu HY, Bai WD, Li C, Zheng Z, Guan H, Liu JQ, Yang XK, Han SC, Gao JX, Wang HT, Hu DH: **Knockdown of lncRNA-ATB suppresses autocrine secretion of TGF-β2 by targeting ZNF217 via miR-200c in keloid fibroblasts.** *Sci Rep* 2016, **6:**24728.

78. Wang Z, Feng C, Song K, Qi Z, Huang W, Wang Y: **lncRNA-H19/miR-29a axis affected the viability and apoptosis of keloid fibroblasts through acting upon COL1A1 signaling.** *J Cell Biochem* 2020, **121:**4364-4376.

79. Tang R, Wang YC, Mei X, Shi N, Sun C, Ran R, Zhang G, Li W, Staveley-O'Carroll KF, Li G, Chen SY: **LncRNA GAS5 attenuates fibroblast activation through inhibiting Smad3 signaling.** *Am J Physiol Cell Physiol* 2020, **319:**C105-c115.
